# The Effectiveness of SBRT for Solitary or Synchronous Multiple Pulmonary Nodules Suspicious of Early-Stage Lung Cancer Without Pathological Confirmation

**DOI:** 10.3390/biomedicines13102534

**Published:** 2025-10-17

**Authors:** Emese Csiki, Dóra Bölcskei, Márton Barabás, Kristóf Gál, Johanna Mikáczó, Szidónia Miklós, Krisztina Trási, Dóra Solymosi, Judit Papp, Mihály Simon, Árpád Kovács

**Affiliations:** 1 Clinic of Oncoradiology, Medical and Health Science Centre, University of Debrecen, 4032 Debrecen, Hungary; 2 Doctoral School of Medicine, University of Debrecen, 4032 Debrecen, Hungary; 3 Department of Oncoradiology, Faculty of Medicine, University of Debrecen, 4032 Debrecen, Hungary; 4 Faculty of Health Sciences, University of Pécs, 7622 Pécs, Hungary

**Keywords:** empirical SBRT, solitary lung nodule, clinically diagnosed lung cancer

## Abstract

**Background**: Stereotactic body radiotherapy (SBRT) is an established curative modality for patients with early-stage non-small cell lung cancer (NSCLC) who are not candidates for surgery. In circumstances where neither surgical resection nor tissue sampling can be performed, SBRT may still be administered empirically, with accumulating evidence indicating excellent efficacy and safety. **Objective**: This single-institution retrospective study aimed to evaluate the clinical outcomes of SBRT for presumed malignant lung lesions, focusing on local control, survival, and treatment-related toxicity, and to compare these findings with published results in histologically confirmed NSCLC. **Methods**: Between 2018 and 2024, 80 cases with 85 pulmonary lesions received SBRT at the Department of Oncoradiology, University of Debrecen. All patients underwent comprehensive staging with chest CT and PET-CT, and treatment decisions were made by a multidisciplinary tumor board. Eligibility required the absence of other primary malignancies within 5 years. Treatment planning was based on 4D-CT imaging with internal target volume delineation across multiple respiratory phases. SBRT was delivered on linear accelerators in 4–8 fractions, to a total dose of 48–60 Gy, using volumetric-modulated arc therapy and daily image guidance with 4D cone-beam CT. **Results**: Most patients presented with solitary lesions, while several had synchronous or metachronous multiple lesions (maximum 3 lesions). The median age was 70.1 years, with 60% ECOG performance status 1. Median follow-up was 21 months. One- and two-year local control rates were 89.8% and 94.3%, respectively, with a 51.4% complete response rate at two years. Mean overall survival was 49.6 months. No grade ≥ 3 toxicities were observed. **Conclusions**: Empirical SBRT is a safe, well-tolerated, and highly effective treatment option in elderly, inoperable patients with presumed malignant lung lesions. Its favorable efficacy supports its broader use as a curative alternative when histological confirmation is not feasible.

## 1. Introduction

Solitary pulmonary nodules (SPNs) that appear clinically malignant require curative treatment. Diagnosis is challenging when tissue sampling is not feasible or inconclusive but imaging suggests a high probability of malignancy. These patients are typically elderly with significant comorbidities. Small malignant-appearing pulmonary lesions are often detected incidentally during screening, as they remain asymptomatic due to their size. Lung screening with low-dose CT (LDCT) significantly reduces lung cancer mortality compared to chest X-ray or no screening [1,2,3]. Therefore, annual LDCT is recommended for high-risk individuals—typically 50–80 years old with at least a 20–30 pack–year smoking history [4,5]. The widespread use of LDCT has increased SPN detection rates up to 51% in high-risk and 13% in general populations [4,6].

A solitary pulmonary nodule is defined as a round, <3 cm lung lesion surrounded by parenchyma without atelectasis or pneumonia [6]. About 1% of detected SPNs are malignant, most commonly adenocarcinomas; metastases may arise from melanoma, colon, breast, or lung tumors. Benign lesions are usually infectious (e.g., tuberculosis and histoplasmosis) or noninfectious (e.g., hamartomas and fibromas) [7]. Radiologically, SPNs may be solid, part-solid, or ground-glass opacities. Growth trend and shape are key for malignancy risk: malignant tumors typically have a volume doubling time of 20–400 days, while stable nodules over 2 years are likely benign [1,7]. Irregular or spiculated borders and upper-lobe location further suggest malignancy [7,8].

The Lung-RADS system classifies nodules into five categories, with Category 4B indicating >15% malignancy risk (e.g., solid nodules >8 mm with growth) [9]. Besides radiological features, clinical factors such as smoking history, COPD, and occupational exposure are integrated into risk models [7]. Validated prediction tools include the Tammemagi model [10], Brock calculator [11], Swensen score [12], and Herder model incorporating FDG-PET data [13]. The 2017 Fleischner Society guidelines recommend follow-up strategies based on malignancy risk—ranging from routine CT monitoring to PET-CT or biopsy in higher-risk cases [8].

PET-CT plays a crucial role in verifying solitary pulmonary nodules, radiotherapy planning, and predicting locoregional or distant metastases. Using 18F-fluorodeoxyglucose (FDG), it supports both diagnosis and treatment monitoring in oncologic and lymphoproliferative diseases. Further evaluation is indicated for nodules > 8 mm [14,15]. PET-CT shows high diagnostic accuracy in SPN, with 95% sensitivity and 82% specificity [16]. However, a low standardized uptake value (SUV < 2.5) does not exclude malignancy, as small or lower-lobe lesions may show reduced uptake due to partial volume or motion effects; carcinoids can also present with low SUV [17]. Specificity is further reduced (≈40%) in regions with endemic infections [18].

PET-CT is valuable in detecting lymph node and distant metastases and often alters treatment decisions; stage modification occurs in up to 42% of NSCLC patients compared with diagnostic CT [19]. For mediastinal nodes, sensitivity and specificity are approximately 81% and 79%, respectively [20]. Enlargement of hilar and mediastinal lymph nodes can be due to benign causes (e.g., inflammation) or malignant causes (such as lymphoma or, more commonly, metastatic lymphadenopathy). The lower specificity of PET-CT can be improved by jointly evaluating metabolic and morphological information. In general, the most commonly used thresholds for determining malignancy in thoracic lymph nodes are: SUVmax > 2.5, short-axis diameter (SAD) > 1 cm, and short-to-long axis ratio (SLR) > 0.7. The combined use of SUVmax and SAD, or SUVmax and SLR in the case of smaller lymph nodes, significantly increases the likelihood of correctly identifying truly malignant nodes [21]. According to ESMO (European Society of Medical Oncology) guidelines, invasive sampling is only recommended after a negative PET-CT if the tumor is >3 cm, central, or associated with enlarged mediastinal nodes (>1 cm) [22]. A delay of ≥10 weeks between PET-CT and stereotactic body radiotherapy (SBRT) significantly worsens locoregional control [23].

During radiotherapy planning, PET-CT improves target delineation, especially in atelectatic lungs, minimizing unnecessary irradiation of adjacent organs [24]. The maximum SUV (SUVmax) can predict treatment response and survival. Radiomic analyses of post-SBRT PET-CT further allow prediction of late regional and distant metastases [25,26,27]. PET-CT is also superior to chest CT in distinguishing post-treatment scarring from recurrence: in 37.3% of NSCLC cases, recurrence was detected only using PET-CT [28]. After SBRT or conventional radiotherapy, a post-treatment SUVmax ≥ 5 strongly predicts recurrence and helps differentiate pseudoprogression from fibrosis [17,29].

Ultimately, a tissue sample is the most appropriate way to determine the etiology of the nodule. This can be fine-needle aspiration or biopsy of the suspicious lesion, depending on its location and accessibility. In cases of peripheral localization, CT-guided transthoracic biopsy (TTB) can be attempted, while bronchoscopic sampling techniques are considered for more central tumors [6].

The gold standard treatment for patients with early-stage non-small cell (NSCLC) lung tumors is surgery, but in approximately 20% of cases, patients are medically inoperable due to other serious cardiac or pulmonary comorbidities [30]. In such cases, stereotactic body radiation therapy (SBRT) may be a curative treatment [31,32]. Stereotactic radiotherapy is a highly precise, modern, and rapidly evolving radiation therapy technique originally developed for the treatment of benign and malignant intracranial lesions. In its advanced form, it is applied to extracranial malignant tumors using a high-precision delivery technique without the need for a traditional stereotactic frame. This method is known as stereotactic body radiotherapy (SBRT). Although various definitions exist in the literature, the 2025 ESTRO (European Society for Radiotherapy and Oncology) SBRT Focus Group provides a clear framework for defining the technique. The essence of SBRT is the delivery of a high biologically effective dose (BED) using image-guided radiotherapy (IGRT) in a hypofractionated regimen to a small, well-defined primary tumor or metastasis, with the goal of achieving local cure. Typically, the fraction dose is ≥5 Gy, and the total number of fractions is ≤8. In the case of lung tumors, the use of organ motion management is also recommended [33].

In inoperable cases, it provides local control equivalent to surgery. Overall survival is close to that of patients who undergo surgery, but does not reach it, given the poorer general condition of the patient group, higher age, and more severe comorbidities [34,35,36]. Operable patients who refuse surgery may be given lung SBRT with good local control and survival outcomes [37,38,39]. It is important to provide patients with the most tolerable local definitive treatment, as the median survival of untreated T1 NSCLC is only 13 months [40].

In our study, we report local control and survival data for early-stage lung cancer patients treated with SBRT at our institute without pathological confirmation and compare them with data reported in the literature.

## 2. Materials and Methods

### 2.1. Patients, Lesions, and Inclusion Criteria

Between 2018 and 2024, 408 patients received lung SBRT at the Department of Oncoradiology, University of Debrecen. Our retrospective study included adult (>18 years) patients who did not undergo histological sampling but were suspected of having malignant pulmonary nodules based on clinical data/imaging studies, and had an ECOG status of a maximum of 2. No pathological diagnosis was made in these patients, which may have been due to the technical impossibility of TTB due to the location of the lung lesion, the patient’s severe comorbidity, or possibly the patient’s refusal to undergo sampling. We did not include patients who had other known primary tumors within 5 years or who had received radiation therapy to the lung within 5 years. Studies on similar topics in the literature generally reported results for 1 lesion/patient [41], whereas we also included patients who underwent SBRT for 2 and then 3 unconfirmed lung lesions. A total of 85 lung lesions underwent elective SBRT during this period, with two synchronous lesions treated with a common plan in 5 cases and a third lesion that appeared later in 3 patients. We irradiated 2 foci separately in 4 patients. In the case of one patient, 3 nodules were treated at different times. Prior to all SBRT, PET-CT was performed for all lesions, which showed no pathological locoregional lymph nodes or distant metastases. Thus, a total of 80 treatments with 85 lesions met the selection criteria. Tumor staging was performed according to the 8th edition of the AJCC (American Joint Committee on Cancer) TNM staging system. A multidisciplinary group (thoracic surgeon, pulmonologist, clinical oncologist, radiation oncologist, radiologist) decided on the appropriate treatment for each patient based on their complete medical history, general condition, and the results of imaging tests and imaging material. Lung SBRT was performed if malignancy was highly likely and surgery could not be performed at the patient’s request or due to the patient’s unfit condition. Based on localization in the lung parenchyma, peripheral and central lesions were included in our study (ultra-central lesions are not treated with SBRT at our institute) [42]. The maximum tumor size was 4.2 cm, clinical T stages T1a-T2b, Stage IA-IIA according to AJCC 8th Edition.

### 2.2. SBRT Treatment Details

For linear accelerator-based lung SBRT treatment, a 4D CT scan was performed on either a Philips Brilliance Big Bore CT (Philips, Amsterdam, The Netherlands) or a SOMATOM go.Sim (Siemens Healthineers AG, Forchheim, Germany) in accordance with the protocol in all cases during spontaneous breathing, and all patients were positioned in a BodyFix BlueBag vacuum mattress. The GTVs (Gross Tumor Volumes) were determined using a lung window and PET-CT fusion on at least 5 of the reconstructed 10 respiratory phases, according to the visible extent of the tumors. Due to possible microscopic spread, no additional margin was added, and the GTV was equivalent to the CTV (Clinical Target Volume). Later, we created an ITV (Internal Target Volume) from the union of the GTVs, thus determining the full movement of the lung lesion during respiration. We formed the PTVs (Planning Target Volume) by adding an institutionally appropriate margin of 5 mm to the ITV, which results from the set-up error. Radiation therapy was delivered using the VMAT technique with 6–10 MV photons in 1 to 2 arcs, and plans were created using Pinnacle (Philips, Amsterdam, The Netherlands) or RayStation (RaySearch Laboratories AB, Stockholm, Sweden) planning systems, taking into account the dose constraints of the organs at risk [42,43,44]. Image Guided Radiotherapy (IGRT) was used prior to all fractions in the form of 4D Cone beam CT (4D CBCT) with physician verification to grant adequate positioning. Depending on the location of the foci (peripheral or central), the prescribed maximum dose was 48 Gy in 4 fractions, 60 Gy in 8 fractions, or 50 Gy in 5 fractions. The goal was to achieve a biologically effective dose (BED) of 100 Gy, calculated with an alpha/beta of 10 Gy. Patients received treatments every other day on a linear accelerator (Elekta Synergy, Versa HD, Elekta AB, Stockholm, Sweden).

### 2.3. Follow up

All patients underwent diagnostic CT or PET/CT scans at least 6 weeks post-treatment. According to the ASCO (American Society of Clinical Oncology) guideline, follow-up of lung tumors is recommended every six months with diagnostic chest CT during the first two years to exclude recurrence, and annually thereafter with low-dose chest CT to detect potential new primary lung cancers. In our study, we evaluated the diagnostic chest CT scans performed at 6 months, 1 year, and 2 years after SBRT. The tumor response to SBRT was determined by measuring the largest tumor diameters on the diagnostic chest CT. PET-CT is not recommended for routine follow-up of lung tumors, but only in cases where recurrence or metastasis is suspected on the performed CT [45]. Taking this into account, and from a cost perspective, PET-CT was performed in only a few cases for the follow-up of our patients. Due to the characteristics of our study population (lack of histological confirmation), PET-CT data could be useful for monitoring metabolic information and for more clearly distinguishing SBRT-induced perilesional pneumonitis/fibrosis compared to chest CT. Local control (LC) was determined based on the results of the available chest CT and PET-CT scans, according to the updated RECIST criteria [46]. We ascertained the six-month, 1-year, and 2-year LC of the lung nodules observed with imaging, and we identified the proportion of non-progressive foci that experienced complete response (disappearance of the target lesion), or partial response (≥30% decrease in the longest diameter of the target lesion) [47]. We examined locoregional progression, the appearance of distant metastases, overall and cancer-specific survival. Follow-up imaging time refers to the time between SBRT and the last chest CT scan. Follow-up duration is the time between the date of the last fraction and the date of the last examination or documented death. We analyzed separately the results of patients who underwent SBRT for only one lesion as well.

### 2.4. Statistical Analyses

Descriptive statistics were used to characterize the patient cohort, including demographic variables (age, gender), baseline clinical features (ECOG performance status, history of COPD, prior tumor history, bronchoscopic and TTB evaluation, operability status), and lesion-specific characteristics (tumor size and SUVmax on pre-SBRT PET-CT, T stage, lobe localization, and central versus peripheral location). Categorical variables were summarized as absolute numbers and percentages, while continuous variables were reported as medians with ranges. Fractionation schedules used in SBRT were also documented and analyzed descriptively. Survival outcomes were assessed using the Kaplan–Meier method, with overall survival estimated from the last day of SBRT to the date of death or last follow-up. Patients alive at last contact were censored at the date of last follow-up.

## 3. Results

Three patients were excluded from the study because they died shortly after SBRT treatment (non-cancer-specific death), even before the first follow-up imaging examination. Overall, the patient group undergoing SBRT was moderately elderly, with a mean age of 70.1 years (range 56–89 years), 37.5% male and 62.5% female. Based on their general condition, 7.5% of patients had an ECOG score of 2. ECOG 1 was the most common condition, occurring in 60% of cases. COPD was present in the medical history of 65% of patients. In 10 patients, we found a primary tumor that had been verified and treated more than 5 years earlier, most commonly bladder, breast, melanoma, and rectal tumors, occurring on average 14.5 years before radiation therapy (5–26 years). Patients with multiple nodules had no history of previous primary tumors. During the investigation of lung lesions, bronchoscopy was performed in 51.3% of cases, and TTB in only 3.80% of cases, to obtain accurate histology. PTX was documented in one case after TTB. The majority of treated patients were inoperable, but 17.5% of all patients were initially operable and refused invasive examination or surgery. In 60 patients, only one lung nodule was treated at a time. The average size of the lesions before SBRT, as determined by PET-CT performed as part of the examination, was 1.9 cm (minimum 0.5 cm; maximum 4.2 cm), based on which the most frequently occurring T stage was cT1b in 48.2% of cases. SUVmax values ranged from a minimum of 0 to a maximum of 28.2 (average 8.5). 51.7% of the lesions were located on the right side, 48.2% on the left side, 55 lesions (64.7%) in the upper lobes, and 25 lesions in the lower lobes. Based on the distance from important mediastinal organs, 94.1% of the lesions were peripheral, and only 5.9% were central, and no lesions in an ultra-central region were treated with stereotactic radiation therapy. Lesions very close to the chest wall (ITV closer than 2 mm to the chest wall) or touching it were present in 44.7% of cases, while 55.3% were located further away from the chest wall. In the 60 single lesion treatments, the average size of the lesions was 2.05 cm, with an average SUVmax value of 8.76; no nodules were located in the right middle lobe (Table 1).

The fractionation scheme used during radiation therapy was 4 × 12 Gy (BED_10_ = 105.6 Gy) in 32 cases, 8 × 7.5 Gy (BED_10_ = 105 Gy) in 43 cases, 7 × 7.5 Gy (BED_10_ = 91.8 Gy) in 7 cases, and 5 × 10 Gy (BED_10_ = 100 Gy) in 1 case. In two treatments, patients received only five and six fractions of the prescribed 60 Gy/8 fractions (not due to an individual problem caused by the lung tumor or side effect of the SBRT). The most commonly used regimen for patients with single and multiple lesions was 60 Gy in eight fractions in 50.6% of cases. In five cases, two lesions were treated simultaneously, and in other cases, one lesion was treated at a time. In three patients, after two lung nodules were treated at the same time, a third single nodule was treated later (Table 1).

The median follow-up time was 21 months (range: 2–56 months). The overall treatment time was 10–17 days, depending on the fractionation regimen. The median follow-up imaging time was 14.5 months. 89.4% of the foci had a SUVmax value higher than 3 on the initial PET-CT scans performed before SBRT as part of the investigation. As part of the follow-up examination after SBRT, PET-CT was performed in only 35 cases, on average 13.8 months after treatment. The SUVmax values in these examinations were 0 in 14 scans, <3 in 19 scans, and >3 in 2 scans.

Six-month local control was interpretable in a total of 66 lesions, 1-year LC in 45 lesions, and 2-year LC in 35 lesions. In the remaining patients, chest imaging (CT, PET-CT) was not performed at the appropriate time, or the CT results were not available to the radiation therapist. Based on these data, the six-month LC was 92.4%, the 1-year LC was 89.8%, and the 2-year LC was 94.3%. A gradual improvement in tumor response was observed over time, with complete response rates increasing from 12.1% at 6 months to 16.3% at 1 year and 51.4% at 2 years, accompanied by a decrease in local enlargement from 7.6% to 10.2% and then 5.7%, respectively. Partial responses were observed in 31.8%, 34.7%, and 22.9% of lesions at 6 months, 1 year, and 2 years, while stable disease was noted in 48.5%, 38.8%, and 20.0% of cases, respectively. Overall, partial and stable responses predominated during the first year, whereas most lesions eventually demonstrated complete radiological remission by the second year (Table 2).

**Figure 1:**
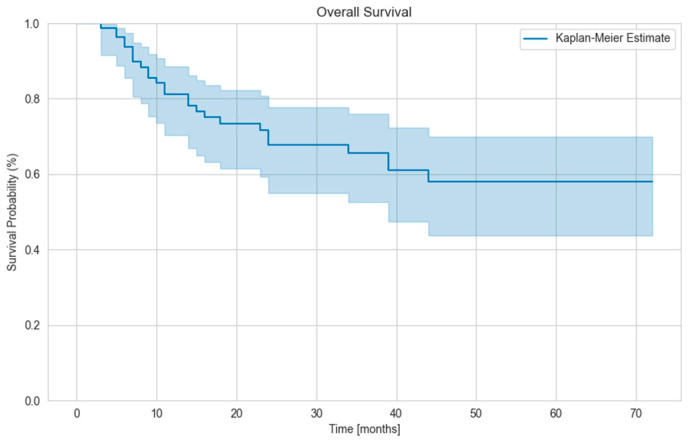
Kaplan–Meier curve for Overall Survival (OS).

**Figure 2:**
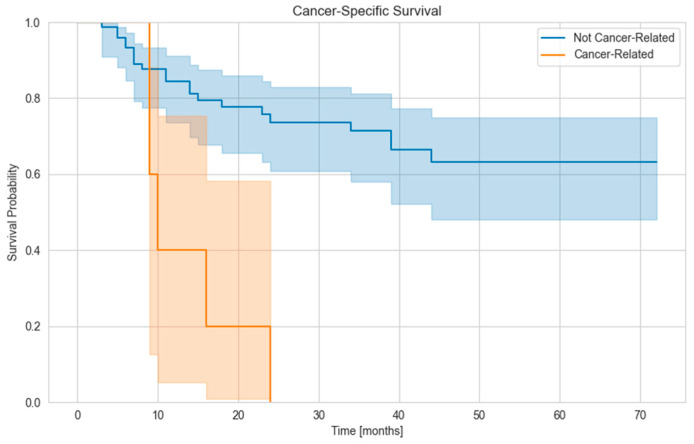
Kaplan–Meier curve for Cancer-Specific Survival (CSS).

Locoregional lymph node metastasis was found in 6 cases and distant metastasis in 20 cases. Distant metastasis developed on average 12.2 months after radiotherapy. The mean OS of the patients was 49,6 months (Figure 1), and the mean cancer-specific survival was 52.6 months. (Figure 2). There was no difference in OS between patients with a single lesion and those with multiple lesions. We examined how many patients had their tumor histology clarified during follow-up (e.g., from newly appearing and more easily accessible metastases). The disease was verified in three patients, with pancreatic adenocarcinoma, bladder tumor, and NSCLC confirmed.

No acute or chronic ≥ grade 3 side effects were documented in the study group, and radiation therapy did not need to be suspended or interrupted in any case due to side effects. We observed one patient whose two pulmonary nodules were treated with SBRT two years apart, and whose ECOG performance status declined from 0 to 2 by the time of the second nodule treatment. This decline was attributable to severe COPD. No ECOG deterioration occurred during radiotherapy, and none of the patients required hospitalization due to the treatment. In one patient, due to dry cough provoked by the supine position (previously known chronic lung disease), we were only able to deliver 5 × 7.5 Gy instead of the prescribed 8 × 7.5 Gy.

We had a few limitations in our retrospective study:

- We excluded patients diagnosed with other primary tumors within 5 years prior to radiation therapy to reduce the chance of treating lung metastases, but in the absence of histological examination, it is possible that we treated metastases in a few cases. Patients with multiple tumors were staged as separate primary tumors. Oligometastatic disease should also be treated locally, and SBRT is an option for these patients [48,49].
- It is difficult to determine the size of recurrence on chest CT scans performed after SBRT, as it can often be confused with pneumonitis or fibrosis caused by radiation therapy [50].
- In many cases, PET-CT was not performed for financial reasons, so pre-treatment SUV max values could not be tracked, except in limited cases.

## 4. Discussion

The primary treatment for small, non-metastatic malignant lung tumors is surgical resection if operable. In inoperable patients, SBRT is a key option for both primary tumors and metastases. The most common histologically confirmed type is NSCLC, for which SBRT is safe and curative [42,51]. SBRT may also be used for limited-stage SCLC when surgery is not possible, though chemotherapy is recommended afterward [52]. In oligometastatic disease, local treatment of a limited number of metastases may include surgery, stereotactic treatment, or interventional radiology, with SBRT as second-line if new lesions or oligoprogression occur [48]. For patients in whom biopsy or surgery is not feasible due to comorbidities, definitive treatment should be offered if imaging strongly suggests malignancy. Given its safety and efficacy comparable to surgery, SBRT is ethically acceptable in these cases [47]. No prospective randomized trials have compared elective surgery, SBRT, and watchful waiting in clinically diagnosed lung cancer.

Compared to small (mean 28.4 mm) malignant-looking lesions and histologically confirmed NSCLC tumors (average 34.2 mm) after SBRT, a 3-year LC rate of approximately 90% can be expected in both cases [53]. Dautruche compared data from 131 clinically and 131 pathologically diagnosed lung tumor patients after SBRT treatment and found no significant differences between the two groups in terms of 3-year LC, 3-year OS, or 3-year regional-distant control [54]. In a Japanese study comparing the results of 115 NSCLC lesions and 58 clinically diagnosed lesions after administration of 5 × 8 Gy or 5 × 10 Gy SBRT, 3-year LC was 87% and 80%, but no significant difference was found in either LC or OS [55].

Several studies have examined this separate group of clinically confirmed lung cancer patients alone. Yoshitake’s retrospective study, with a median follow-up time of 23 months, analyzed data from 88 patients, and after 4 × 12 Gy SBRT, the 3-year LC was 90%, and subgroup analysis using univariate analysis found the baseline SUVmax value to be a significant prognostic factor [56]. According to a retrospective study conducted at the University of Louisville by Harkenrider et al., regardless of whether infectious granulomatous disease is endemic in this region (southeastern US), if a malignant lung lesion is likely based on PET-CT (PET-CT positive predictive value 86%), patients benefit from SBRT treatment, with estimated local control exceeding 97% and no grade 3 acute side effects [57]. After SBRT of small clinically diagnosed lung lesions, comparing lesions smaller than 2 cm with larger lesions, significantly higher OS and regional-distant control were observed after treatment of lesions smaller than 2 cm, with no difference in LC [58]. An early (2004–2012) retrospective analysis of more than 7000 patients with T1a-T2a lung tumors showed that clinical diagnosis may be associated with better cancer-specific survival (CSS), but there was no difference in OS. According to the registry serving as the source of patient data, 91.2% of patients who underwent radiation therapy had a pathological diagnosis, while in 9.2% no tissue sample was taken. As reported by the authors, the improved CSS may be due to the higher proportion of small (<2 cm) tumors in the clinical diagnosis group, which may have concealed benign lesions [59]. Fan and his team reported long-term results (median follow-up time 69 months) for early-stage lung cancer patients treated with SBRT using helical tomotherapy, showing no significant difference in 5-year LC, OS, and CSS between histologically verified and unverified NSCLC patient groups [60]. Wang et al. summarized the results of lung SBRT with Cyberknife (median BED: 136 Gy) in twenty-five patients over the age of 75. LC was examined as the primary endpoint, with 1-year and 3-year LC rates of 100% and 78.8%, respectively, and grade 3 pneumonitis, an acute side effect, occurred in a total of two patients [61]. A Dutch population-based analysis of elderly patients with early-stage lung cancer (aged 75 years or older) examined the impact of the introduction and spread of SBRT (between 2001 and 2009) on survival. This new, effective treatment method, which can also be used in elderly patients, has reduced the number of patients who do not receive curative treatment (7% absolute reduction) and, at the same time, significantly improved the survival of patients receiving SBRT [62]. Based on a retrospective analysis of patients over 80 years of age with stage I NSCLC following SBRT treatment (65 foci examined), 1-year LC and OS were 98.4% and 88.9%, respectively. Five-year results were also reported, with LC: 87.4% and OS: 47.5%. The prescribed dose was 4 × 12 Gy, with 10 × 6 Gy in approximately 10% of cases [63].

Elective stereotactic treatment plays a decisive role in cases of lung tumors that have not been verified by histology. 90% of untreated patients with early-stage NSCLC die within 5 years, while this number is even higher for small cell lung cancer (SCLC) patients [30]. There are no clear guidelines available, but given the large number of patients, definitive treatment should be chosen when there is a well-founded suspicion of a malignant lung lesion based on imaging studies. A meta-analysis published in 2014 (which compared the long-term outcomes of surgical and SBRT treatments for stage I NSCLC) shows that elective SBRT was also performed in the majority (>50%) of selected lung SBRT studies, and approximately 65% of the lesions were not histologically verified [34,60].

We found that, consistent with data reported in the literature, the 1- and 2-year local control (LC) rates of the treated pulmonary lesions were 90–95%. In our study, interestingly, the complete response rate increased to 51.43% at 2-year follow-up (1-year complete response rate was 16.33%), which may be because after 2 years, pseudoprogression caused by radiation treatment can be clearly distinguished, and soft tissue density can disappear if there is no tumor anymore.

In a systematic review, the possible radiographic changes observable on chest CT following lung SBRT were summarized. Compared to conventional 3D conformal radiotherapy, different post-treatment alterations may appear after stereotactic irradiation of the lung, as a steep dose gradient develops adjacent to the tumor (target volume), and a large volume of normal lung tissue receives a low radiation dose. In such cases, a mass-like, space-occupying appearance may develop, which can be misinterpreted as tumor progression. The radiologic changes can represent acute pneumonitis (<6 months after SBRT), most commonly presenting as consolidation, or late fibrotic alterations (>6 months after SBRT), such as mass-like fibrosis [29]. Most studies indicate that definite local recurrence or tumor progression should be suspected when the consolidation increases in size more than 12 months after SBRT, often accompanied by additional CT findings such as the disappearance of the air bronchogram [64]. When recurrence is suspected, PET-CT is recommended; a SUVmax greater than 5 suggests a higher likelihood of malignancy [29]. Tumor progression should be determined according to RECIST 1.1 criteria, although accurate lesion size assessment may be challenging in cases of semi-solid or ground-glass nodules [65]. Following lung SBRT, consolidation develops in approximately three-quarters of cases, but only about one-third of these cases represent true tumor progression [66].

## 5. Conclusions

It is important that, where possible, the histology of the tumor under investigation is determined and that the appropriate treatments are selected with this in mind. Accurate histology is particularly important in the progression of the disease and in the assessment of systemic treatments (chemotherapy, immunotherapy).

However, in patients in whom sampling is not feasible due to other morbidities or poor general condition, and clinically malignant lesions are confirmed in the lung, appropriate curative treatment should be given even without histology. Based on our results, it is clear that if histology cannot be obtained from one or more (maximum three) lung lesions suspected of malignancy, given the poor prognosis of the malignant lesions, SBRT should be given as definitive treatment, which is effective, safe, and well tolerated, even in elderly patients who are not in good physical condition.

## Figures and Tables

**Table 1:** Baseline patient, lesion, and treatment characteristics.

		All of the Treatments	One Lesion Treatments
		Patients (n = 80):	Patients (n = 60):
**Age (y):**			
	Range	56–89	58–89
	Mean	70.1	70.7
**Gender—male/female (%):**		37.5/62.5	45/55
ECOG * (%):			
	0	32.5	33.3
	1	60	61.7
	2	7.5	5
COPD ** (%):		65	63.3
**Primary tumor earlier (%):**		12.5	16.7
**Bronchoscopy (%):**		51.3	55
TTB *** (%):		3.8	5
**Operability (%):**		17.5	23.3
		Lesions (n = 85)	Lesions (n = 60)
**Tumor size (cm):**			
	Range	0.5–4.2	0.7–3.7
	Mean	1.9	2.05
**T stage (%):**			
	cT1a	14.1	8.3
	cT1b	48.2	48.3
	cT1c	23.5	26.7
	cT2a	12.9	16.7
	cT2b	1.2	0
SUVmax ****:			
	Range	0–28.2	1.9–28.2
	Mean	8.5	8.76
**Tumor location:**			
**Lung lobes (%):**			
	Left upper lobe	36.5	38.3
	Left lower lobe	11.8	10.0
	Right upper lobe	28.2	31.7
	Right middle lobe	5.9	0.0
	Right lower lobe	17.6	20.0
**Central/peripheral (%):**		5.9/94.1	5.0/95.0
**Near chest wall (%):**		44.7	46.7
**Fractionation scheme (%):**			
	8 × 7.5 Gy	50.6	58.3
	4 × 12 Gy	37.6	36.7
	7 × 7.5 Gy	8.2	5
	5 × 10 Gy	1.2	0

* Eastern Cooperative Oncology Group Performance Status Scale. ** Chronic Obstructive Pulmonary Disease. *** Transthoracic Biopsy. **** Maximal Standard Uptake Value.

**Table 2:** Local Control (LC) data at 6 months, 1 year, and 2 years. Treatment response is described as follows: complete response (disappearance of the target lesion), partial response (≥30% decrease in tumor size), local enlargement (≥20% increase in tumor size), and stable disease (<30% decrease or <20% increase in tumor size).

		[%]
**LC @ 6 months:**		92.40%
	Complete response	12.12%
	Partial response	31.82%
	Stable Disease	48.48%
	Local Enlargement	7.58%
**LC @ 1 year**		89.80%
	Complete response	16.33%
	Partial response	34.69%
	Stable Disease	38.78%
	Local Enlargement	10.20%
**LC @ 2 years**		94.30%
	Complete response	51.43%
	Partial response	22.86%
	Stable Disease	20.00%
	Local Enlargement	5.71%

## Data Availability

The data presented in this study are available from the corresponding author due to ethical reasons upon request.
